# The Relationship Between Drivers’ Cognitive Fatigue and Speed Variability During Monotonous Daytime Driving

**DOI:** 10.3389/fpsyg.2018.00459

**Published:** 2018-04-04

**Authors:** Jinfei Ma, Jiaqi Gu, Huibin Jia, Zhuye Yao, Ruosong Chang

**Affiliations:** ^1^School of Psychology, Liaoning Normal University, Dalian, China; ^2^Key Laboratory of Child Development and Learning Science, Ministry of Education, Research Center for Learning Science, Southeast University, Nanjing, China

**Keywords:** cognitive fatigue, vigilance, speed variability, self-regulation, simulated driving, fatigue countermeasures

## Abstract

A lack of task workload can lead to drivers’ cognitive fatigue and vigilance decrement during a prolonged drive. This study examined the effects of speed variability on driving fatigue in a monotonous drive. Twenty-one participants participated in a 60-min simulated driving task. All participants’ cognitive fatigue was assessed using psychological and physiological measurements. Results showed that among all participants, variability of vehicle speed was negatively correlated with sleepiness and hypo-vigilance during the driving task. Further, drivers in the large variability group reported less sleepiness, less fatigue, and more vigilance than those in the small variability group did during the driving task. These drivers also presented a smaller electroencephalogram spectral index (𝜃+α)/β during the task, where 𝜃, α, and β are the power spectra of three different frequency bands: theta (𝜃, 4∼8 Hz), alpha (α, 8∼13 Hz), and beta (β, 13∼30 Hz). Our findings suggested that the larger variability of speed within the speed limit may have a deterrent effect on drivers’ cognitive fatigue.

## Introduction

Over 1.2 million people are killed in the traffic accidents each year around the world, with millions of people suffering from severe injuries and living with long-term adverse health consequences (World Health Organization, 2015). Hypo-vigilance and fatigue have been regarded as the significant contributors for road accidents. Empirical studies have reported that approximately 16–23% of car crashes on the highways in southwest and midland England and 21.9% in Italy were caused by sleepiness or fatigue (Horne and Reyner, 1995; Garbarino et al., 2001). Albeit precise contributors of these factors have not yet been determined in crashes, researchers have reached a consensus that fatigue represents one major road safety hazard. Therefore, it is urgent to develop effective coping strategies for management of driver fatigue.

Driving is considered a vigilant task, in which the driver needs to maintain a high level of alertness. May and Baldwin (2009) suggested that drivers’ cognitive fatigue could be either caused by circadian rhythm and sleep disturbance (i.e., sleep-related fatigue) or caused by cognitive overload or underload (i.e., task-related fatigue). For task-related fatigue, cognitive overload commonly is induced by high task demands requiring sustained attention; however, prolonged driving that offsets driver workload can benefit such form of task-related fatigue. In contrast, cognitive underload is induced by continuous and monotonous driving conditions, which require an increased task novelty and demand to reduce this form of task-related fatigue (Hancock and Warm, 1989; Thiffault and Bergeron, 2003; Saxby et al., 2013). Besides, a previous study has concluded that fatigue induced by cognitive underload would impair a driver’s engagement more rather than that induced by cognitive overload (Saxby et al., 2008). Once the demands of driving become more familiar and monotonous, the driver is more vulnerable to reach an underload state. According to the theory of attentional resource shrinkage (Young and Stanton, 2002), an underload state could keep the arousal at a low level, and lead to vigilance decrements as well as poor driving performance. Thus, a monotonous prolonged drive requires more attentional resources that cause driver vigilance. On the other hand, Hockey’s compensatory control model (Hockey, 1997) proposed that we constantly regulated our effort based on the relative importance of goals we had, and changes in mental effort were representative of task difficulty. Therefore, a monotonous driving task represents reduced task effort, causing boredom and fatigue.

Further, a study found that drivers’ speed variability became larger when the monotonous drive lasted for over 80 min (Gershon et al., 2009). They attributed this phenomenon to the driver’s speed control capability becoming worse in the state of cognitive fatigue. However, the task-capability interface model suggested that the choice of vehicle speed could be influenced by the driver’s cognitive workload (Fuller, 2005). Drivers changed their speed maybe in order to adjust the task difficulty actively and increase their arousal levels. In this study, we investigated the effect of drivers’ variability of vehicle speed (within the speed limit) on their cognitive fatigue. Currently, neurophysiological approaches, like electroencephalogram (EEG) and eye tracking, are widely employed for evaluating driving cognitive fatigue (Borghini et al., 2014). Literature has proved that EEG and pupil diameter can be used as a physiological index for monitoring the level of drivers’ vigilance (Makeig and Jung, 1995; Jap et al., 2009; Zhao et al., 2012; Heeman et al., 2013; Pfleging et al., 2016). In particular, the fluctuations in certain EEG band power are sensitive to the vigilance level (Zhao et al., 2012). For example, increased EEG alpha power may indicate the vigilance decrement (Lal and Craig, 2002). Therefore, we employed the psychophysiological methods (e.g., subjective fatigue state, EEG index, and eye movements) to measure driver’s cognitive fatigue during a prolonged simulator-driving task.

## Materials and Methods

### Participants

This study was approved by the Research Ethics Committee of Liaoning Normal University in December 2016. Participants were recruited via flyers distributed in the local taxi companies in Dalian, China, from December 2016 to January 2017. We approached 31 professional drivers by phone. Eventually, 21 healthy male participants (mean age: 40.1 years, age range: 29–47 years), who are licensed drivers, were recruited. All participants reported having a full license for an average of 15.8 years, driving more than 100,000 km in a typical year, and having no prior experiences of driving in a simulator. Participants were instructed to go to bed no later than10 P.M. before the experiment, and to get up around 7 A.M. on the experimental day. The experiment started at 9 A.M. It was also ensured that all participants refrained from consuming caffeine or alcohol in the morning of their visits. Participants who had not had enough sleep (e.g., less than 8 h) were ruled out from the experiment. All participants gave their written informed consents before the experiment and were offered a monetary compensation of 100 RMB at the end of the study.

### Procedure

This experiment took place in a soundproof lab of the university. When arrived at the laboratory, participants were given a full instruction of the experiment by the experimenter and a trial drive in the simulator. Then, they were connected to the EEG and eye tracker monitors for the recording of physiological data and completed a 60-min driving task. Before and after the driving simulation task, participants were required to complete the questionnaires to assess their subjective fatigue state. In addition, each participant’s vigilance level was evaluated by the vigilance measurement at the end of the driving task.

In order to create the monotonous task, participants were instructed to follow the former vehicle (without overtaking) and to keep the minimum following distance at 100 m. The lead vehicle traveled at a constant speed of 70 km/h and could stop randomly during the experiment. Therefore, participants were required to maintain a safe distance for preventing a rear-end collision. During the experiment, the average driving speed among all participants was 72 km/h, which was consistent with that of the lead vehicle.

### Measures

#### Subjective Measures

Perceived fatigue induced by the driving task was assessed using the Chinese version of Swedish Occupational Fatigue Inventory (SOFI-C) with a 0–10 numerical response scale (Åhsberg et al., 1997), which has a good validity and reliability in China (Lin et al., 2012). The questionnaire consists of 25 questions describing five dimensions: physical discomfort, physical exertion, lack of energy, lack of motivation, and sleepiness. In addition, a retrospective assessment of the driver’s vigilance level with a 1–9 numerical rating scale was used (**Table 1**). This measurement consists of four items, which modify the Karolinska Sleepiness Scale (KSS) (Åkerstedt and Gillberg, 1990), inattention (ATT), and monotony (MON) (Schmidt et al., 2009), to assess each participant’s vigilance level, including the aspects of boredom, sleepiness, inattention, and monotony with regard to the whole driving.

**Table 1:** Measurement of the drivers’ vigilance level.

Concerning the period of the whole drive									
1	…how would you describe your predominant state?								
	Extremely alert		Alert		Neither alert nor sleepy		Sleepy, but no difficulty remaining awake		Extremely sleepy, fighting sleep
	1	2	3	4	5	6	7	8	9
2	… how attentively have you been driving?								
	Extremely attentively		Attentively		Neither attentively nor inattentively		Inattentively		Extremely inattentively
	1	2	3	4	5	6	7	8	9
3	… how did you perceive the drive?								
	Extremely varied		Varied		Neither varied nor monotonous		Monotonous		Extremely monotonous
	1	2	3	4	5	6	7	8	9
4	… how did you feel about the driving task?								
	Extremely interesting		Interesting		Neither interesting nor boring		Boring		Extremely boring
	1	2	3	4	5	6	7	8	9

#### Performance Measures

**FIGURE 1:**
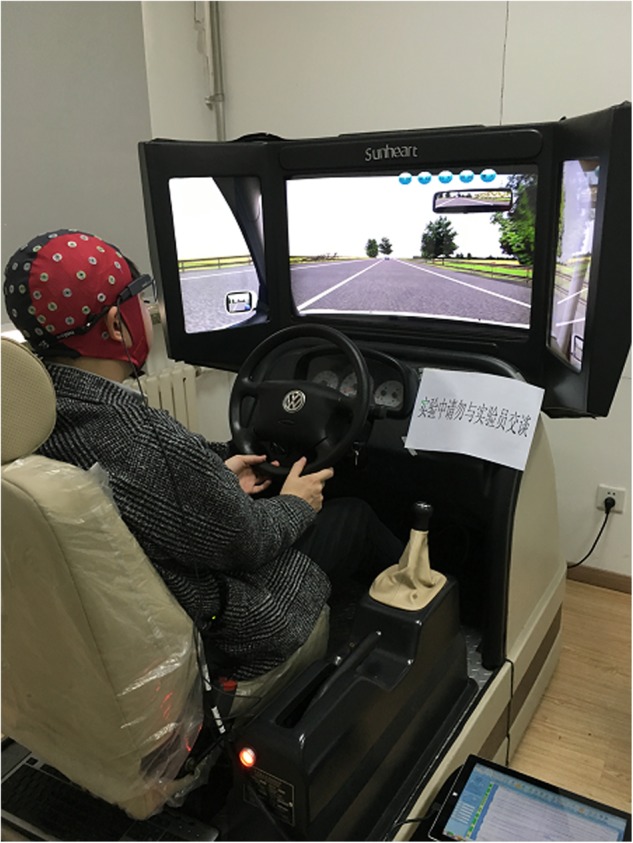
The road scene of the driving simulator.

A fixed-based car driving simulator (QJ-3A1; Beijing Sunheart Simulation Technology, Co., Ltd.) was employed in this study. The road scene of the driving simulator used in the experiment was set as a monotonous highway condition, as shown in **Figure 1**. The driving scenario was a two-lane closed track, 5.25 km long, with 10 curves. Driving performance measures including standard deviation (SD) of lane position, mean and SD of speed, and car following distance were recorded during the whole driving task.

Considering that the adaptation period to the driving simulator was about 15.4 min in the curved-road scenarios and about 12.1 min in the straight road scenarios (Ronen and Yair, 2013), we defined the first 15 min of the driving test as the adaptation period. Therefore, the whole driving test session (considered as the dependent variable) was split into three segments with 15 min per segment: segment 1, segment 2, and segment 3. Each participant was required to drive three runs per segment under the experimental road condition.

#### Eye Movement Measures

Eye movement data were recorded using the head-mounted Tobii Glasses II eye tracking system (Tobii, Sweden), which allowed free movement of the head. The sample frequency of the eye tracker was 50 Hz with a 82° × 52° recording visual angle.

#### EEG Measures

Eelectroencephalogram signals were recorded continuously using a portable 63-channel ‘eego amplifier’ EEG system (eegoTMmylab, ANT B.V., Netherlands) with an extended 10–20 system layout. The sensor net was aligned with respect to three anatomical landmarks including two pre-auricular points and the nasion. Impedances were kept below 10 kΩ, and the sampling rate was 1000 Hz. The electrode CPz was used as the reference, whereas, the electrode AFz was used as the ground.

### Data Analysis

#### Eye Movement Data Preprocessing

Eye movement measures involved three indices: pupil diameter (unit: mm) and horizontal and vertical positions of each gaze (unit: pixels). The positions of each gaze were rotations around the horizontal axis and the vertical axis.

#### EEG Data Preprocessing

Electroencephalogram data were pre-processed using EEGLAB (Delorme and Makeig, 2004), an open source toolbox running in the MATLAB (v.2010a; The MathWorks, Inc., United States) environment, and in-house MATLAB functions. Continuous EEG data were band-pass filtered between 0.5 and 30 Hz and the sampling rate decreased to 250 Hz. EEG data were referenced to the average of both mastoids (M1, M2). EEG data were removed 30 s before and after a braking action, due to the huge movement of the participant. Data portions contaminated by eye movements, electromyography, or any other non-physiological artifacts were corrected using the Independent Component Analysis algorithm (Makeig et al., 1997; Jung et al., 2001). Then the pre-processed continuous EEG data were segmented into dozens of epochs, with an epoch length of 2000 ms. EEG epochs contaminated by strong muscle artifacts or with amplitude values exceeding ±100 μV at any electrode were manually rejected.

To ensure the ecological validity of our experiment, this study did not strictly control participants’ head movements during the task. Therefore, participants having poor quality of EEG data (<80% good data) would be excluded. As a result, all datasets were available for the power spectral analysis.

#### EEG Power Spectrum Estimation

For each participant, the segmented EEG epochs were subjected to the power spectral density analysis by fast Fourier transforms (50% Hamming window) and were exported for analysis in a frequency resolution of 0.5 Hz (range: 0.5–30 Hz). The power spectrum of each frequency point was averaged over the epochs. Then the power spectrum of four frequency bands was computed as the mean value within each frequency limit: delta (δ, 0.5∼4 Hz), theta (𝜃, 4∼8 Hz), alpha (α, 8∼13 Hz), and beta (β, 13∼30 Hz) waves. Previous research suggested that the increased EEG algorithm (𝜃+α)/β was an effective indicator for detecting drivers’ fatigue (Jap et al., 2009). Therefore, this ratio was chosen as a factor that assessed drivers’ fatigue state.

**FIGURE 2:**
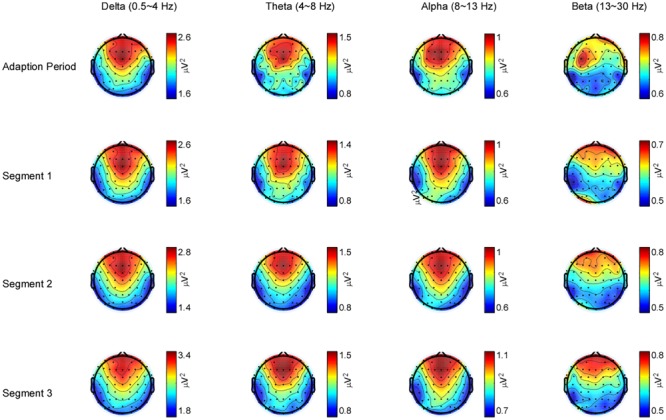
Electroencephalogram (EEG) power spectra for frequencies between 0.5 and 30 Hz during the driving task. Scalp topographies are displayed at the greatest power of delta (0.5∼4 Hz), theta (4∼8 Hz), alpha (8∼13 Hz), and beta (13∼30 Hz) bands for the adaption period, segment 1, segment 2, and segment 3. The delta, theta, and alpha band power shows a positive maximum over the fronto-central electrodes and a negative maximum over bilateral parietal electrodes in each segment, whereas the beta band power shows a positive maximum over the frontal electrodes and a negative maximum over bilateral parietal electrodes.

According to the scalp topography of each band power, the largest power of delta, theta, alpha, and beta bands were consistently shown in the frontal, central, and parietal lobes during the whole driving task, and the power of each band was distributed symmetrically on bilateral hemispheres. Meanwhile, the artifact in the frontal lobe was larger than that in other scalp regions, as shown in **Figure 2**. Therefore, this study chose data from C1, C2, CP1, CP2, P1, and P2 electrodes for further statistical analysis.

### Statistical Analysis

In this study, Pearson’s correlation analysis was employed to investigate correlations between changes of subjective fatigue (i.e., post-task–pre-task), vigilance level, EEG data, driving performance, and eye movement data. Thereafter, all participants were divided into two groups, according to the participant’s driving speed variability. The average speed variability among all participants was 19.67 km/h (*SD* = 2.36 km/h). Eventually, there were 11 participants in the small speed variability group (speed variability = 17.63 km/h), whereas there were 10 participants in the large speed variability group (speed variability = 21.40 km/h). This study adopted the student’s independent-samples *t*-test to analyze the group differences of the subjective fatigue and the drivers’ vigilance level. Driving performance, eye movement, and EEG data were analyzed using a 2 (speed variability) × 3 (driving session) repeated-measures analysis of variance (ANOVA). In order to reduce the risk of a type one error in multiple comparisons, Bonferroni-Šidák’s adjustments of *p*-values were applied. A 95% confidence level was employed throughout. Statistical analyses were carried out using the SPSS 18.0 statistical analysis package (*p* ≤ 0.05; SPSS, Inc., Armonk, NY, United States).

## Results

### Correlations Between Variables

**Table 2** shows correlations between all measures among all participants. Among all participants, variability of speed (i.e., average of SD of speed during the whole drive) was significantly negatively correlated with sleepiness [*r*(19) = -0.50, *p* < 0.05], and vigilance level [*r*(19) = -0.53, *p* < 0.05]. Results suggested that participants with greater speed variability reported to be less sleepy and more vigilant after the monotonous driving task. Among all participants, the pupil diameter (during segment 3) was significantly negatively correlated with sleepiness [*r*(19) = -0.62, *p* < 0.01], total fatigue [SOFI; *r*(19) = -0.46, *p* < 0.05], and vigilance level [*r*(19) = -0.45, *p* < 0.05]. Results indicated that participants having larger pupil diameters during segment 3 of the driving task reported to be less sleepy, less tired, and more vigilant. Among all participants, the EEG index (during segment 3) was significantly positively correlated with total fatigue [*r*(19) = 0.55, *p* < 0.05], lack of energy [*r*(19) = 0.58, *p* < 0.01], lack of motivation [*r*(19) = 0.48, *p* < 0.05], and sleepiness [*r*(19) = 0.58, *p* < 0.01]. Results indicated that participants showing greater EEG activities reported to have less energy and less driving motivation and to be more tired and more sleepy after a prolonged drive.

**Table 2:** Correlations for measurements of standard deviation of vehicle speed, each dimension and total scale of the SOFI (post–pre test scores), vigilance level (post-test scores), EEG algorithm (𝜃+α)/β (segment 3), and pupil diameter (segment 3).

Variables	1	2	3	4	5	6	7	8	9
(1) SD of speed	–								
(2) LOE	–0.23	–							
(3) PE	–0.11	0.67**	–						
(4) PD	–0.17	0.69**	0.82**	–					
(5) LOM	–0.35	0.76**	0.53*	0.74**	–				
(6) Sleepiness	–0.50*	0.68**	0.31	0.49*	0.72**	–			
(7) SOFI	–0.33	0.90**	0.77**	0.88**	0.90**	0.77**	–		
(8) Vigilance	–0.53*	0.59**	0.56**	0.46*	0.50*	0.60**	0.64**	–	
(9) Pupil diameter	0.27	–0.38	–0.30	–0.27	0.34	–0.62**	–0.46*	–0.45*	–
(10) EEG (𝜃+α)/β	–0.30	0.58**	0.23	0.41	0.48*	0.58**	0.55*	0.34	–0.32

*^∗^*p* < 0.05; ^∗∗^*p* < 0.01. SD of speed, standard deviation of speed; LOE, lack of energy; PE, physical exertion; PD, physical discomfort; LOM, lack of motivation; SOFI, Swedish Occupational Fatigue Inventory.*

### Subjective Measures

Among all participants, significant differences were found in all five dimensions and the total scale of the SOFI (*p* < 0.05), where the pre-task scores were significantly lower than the post-task ones. For the group difference analysis, the student’s independent-samples *t*-test showed that there were no significant differences in pre-task scores on each dimension and neither on the total scale of the SOFI between two groups with different speed variabilities (*p* > 0.05; **Table 3**). The student’s independent-samples *t*-test, by assessing the changes (i.e., post-task–pre-task) on each dimension and on the total scale of the SOFI, showed that they were of statistical significance between groups when looking at sleepiness [*t*(19) = 2.10; *p* = 0.05; η^2^= 0.188] as well as of marginal significance in total fatigue [*t*(19) = 1.89; *p* = 0.07; η^2^ = 0.159]. To be specific, participants with large variability of vehicle speed had smaller changes in sleepiness and total fatigue than those with small variability of vehicle speed. Besides, there were significant differences between groups in vigilance level [*t*(19) = 2.32; *p* < 0.05; η^2^ = 0.221] after a prolonged drive, indicating that the large speed variability group was more vigilant during a monotonous drive. However, there were no statistically significant group differences in lack of energy, physical exertion, physical discomfort, and lack of motivation (*p* > 0.05).

**Table 3:** The descriptive data on the scale of SOFI and vigilance level (*M ± SD*).

	Small speed variability group (*n* = 11)		Large speed variability group (*n* = 10)	
	Pre-test	Post-test	Pre-test	Post-test
Lack of energy	1.24 ± 1.88	4.18 ± 2.47	0.26 ± 0.75	1.98 ± 2.49
Physical exertion	0.76 ± 0.62	2.73 ± 2.32	0.60 ± 1.05	1.52 ± 1.83
Physical discomfort	0.49 ± 0.78	3.31 ± 3.14	0.42 ± 1.13	1.52 ± 1.73
Lack of motivation	1.82 ± 2.05	5.00 ± 2.17	0.76 ± 1.61	2.42 ± 2.46
Sleepiness	1.62 ± 2.50	4.89 ± 2.56	0.68 ± 1.23	1.86 ± 1.54
SOFI	1.19 ± 1.38	4.02 ± 2.35	0.54 ± 1.06	1.86 ± 1.87
Vigilance	–	6.45 ± 1.81	–	4.70 ± 1.64

*SOFI, Swedish Occupational Fatigue Inventory.*

### Speed Variability

For all participants, the one-way repeated measures ANOVA (four driving segments: adaptive period and three driving sessions) showed that there was no significant main effect of driving segment [*F*_(1,19)_ = 1.23, *p* > 0.05; **Table 4**]. This result suggested that participants showed stable speed variability during the whole driving task. After splitting all participants into two groups (i.e., small vs. large speed variability), the two-way repeated measures ANOVA showed that there was a significant main effect of groups during the whole task [*F*_(1,18)_ = 20.92, *p* < 0.001, η^2^= 0.538; **Table 4**]. The main effect of the driving segment and its interaction with the group were not statistically significant [*F*_(1,18)_= 1.25, *p* > 0.05]. Analysis of the SD of lane position, average of speed, and car following distance showed no significances of main effects or interactions between group and driving segment (*p* > 0.05).

**Table 4:** Variability of vehicle speed in the adaptive period and three driving sessions (*M ± SD*).

	Adaption period	Segment 1	Segment 2	Segment 3
All participants (*n* = 21)	20.06 ± 2.79	18.89 ± 3.43	19.14 ± 2.20	18.84 ± 3.97
Small speed variability group (*n* = 11)	18.45 ± 2.76	17.00 ± 3.44	18.27 ± 2.23	16.82 ± 3.35
Large speed variability group (*n* = 10)	22.02 ± 1.06	21.20 ± 1.48	20.20 ± 1.71	21.31 ± 3.29

### Physiological Measures

#### EEG Index

**FIGURE 3:**
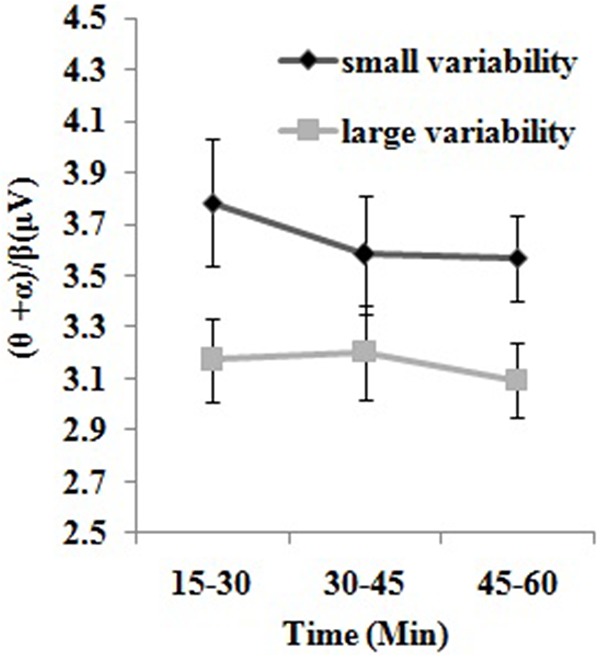
Comparisons of the EEG index between participants with small vs. large variability of speed in three driving segments. Three 15-min driving segments respectively correspond to segment 1, segment 2, and segment 3. Small variability represents participants with small speed variability, whereas large variability represents participants with large speed variability. Data from small and large speed variability groups are marked in black and gray, respectively. Error bars represent the standard errors of the means.

Our 2 × 3 experimental design used the variability of vehicle speed (i.e., small vs. large speed variability group) as the independent variable and driving segment (i.e., segment 1, segment 2, and segment 3) as the dependent variable. As shown in **Figure 3**, a two-way repeated measures ANOVA on the EEG algorithm (𝜃+α)/β yielded a significant main effect of the groups [*F*_(1,19)_ = 4.79, *p* < 0.05, η^2^= 0.201]. Results indicated that participants in the small speed variability group had a greater EEG activity than those in the large speed variability group. The main effect of driving segment and interactions between group and driving segment were not statistically significant (*p* > 0.05). In addition, the student’s independent-samples *t*-test showed that there was no significant difference in EEG data between two groups during the adaption period (*p* > 0.05), suggesting that participants in two different speed variability groups had similar fatigue state at the beginning of the driving task.

#### Eye Movement Data

**FIGURE 4:**
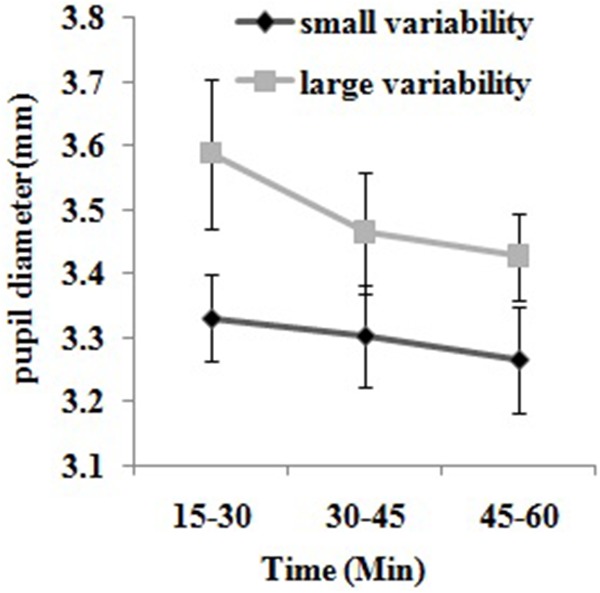
Comparisons of the pupil diameter between participants with small vs. large variability of speed in three driving segments. Three 15-min driving segments respectively correspond to segment 1, segment 2, and segment 3. Small variability represents participants with small speed variability, whereas large variability represents participants with large speed variability. Data from small and large speed variability groups are marked in black and gray, respectively. Error bars represent the standard errors of the means.

As shown in **Figure 4**, a two-way repeated measures ANOVA on pupil diameter yielded the significant main effect of driving segment [*F*_(1,19)_ = 4.79, *p* < 0.05, η^2^= 0.201], showing that all participants’ pupil diameters became smaller as the driving duration increased. However, the main effect of variability of vehicle speed and its interactions with driving segment were not statistically significant (*p* > 0.05). In addition, the Student’s independent-samples *t*-test showed that there was no significant difference in the pupil diameter between two groups during the adaption period (*p* > 0.05). Horizontal and vertical spread of attention showed no significance of main effects or interactions between group and driving segment (*p* > 0.05).

## Discussion

In this study, the psychological and physiological data confirmed that the 60-min prolonged monotonous driving task successfully elicited driver’s cognitive fatigue, indicating an underload state (Desmond and Hancock, 2001). In addition, drivers’ speed variability was associated with vigilance decrement and cognitive fatigue. Specifically, drivers with large speed variability, compared with those with small speed variability, had less sleepiness, more vigilance, and a smaller EEG algorithm (𝜃+α)/β.

Different from previous studies (Charlton and Starkey, 2011, 2013), we observed that the drivers’ speed variability was not significantly different in different driving segments. This may be due to the short driving period (1 h in total), during which the drivers’ speed variability did not decline significantly. Indeed, our study demonstrated stable individual differences of driver’s speed variability, which could last for the whole driving task. Thus, our results confirmed that the driving speed preference varied among individuals.

Our study found that during the driving task, drivers with smaller pupil diameter displayed less sleepiness, less fatigue, and more vigilance, whereas, those with stronger EEG activities had less energy, less driving motivation, and more fatigue. It has been well-known that cognitive fatigue may induce alterations of the EEG band power (Zhao et al., 2012). These neurophysiological indicators supported the dynamic model of stress and sustained attention, claiming that a monotonous drive can induce an underload state (Hancock and Warm, 1989). Our findings also reported that drivers with larger speed variability were less sleepy and more vigilant during a monotonous drive. Larger speed variability indicates a larger degree of changes in driving speed, which requires more attentional resources and increases mental workload. Additionally, driving behaviors, like speed variability, are sensitive to a driver’s alertness state (Ghosh et al., 2015). According to Hancock and Warm’s (1989) theory, increasing the workload can help drivers maintain the optimal arousal level. Therefore, our findings may suggest that driving behaviors, in particular speed variability, may have positive impacts on cognitive fatigue in an underload condition.

Cognitive fatigue is considered to be associated with vigilance decrement. In our study, the large speed variability group reported less sleepiness and more vigilance, compared with the group with small speed variability. Psychological results further supported that drivers with large speed variability may keep themselves in an optimal arousal level during a monotonous drive, thereby reducing driving fatigue. Furthermore, our neurophysiological data showed that drivers who had a large variability of vehicle speed showed a significantly smaller EEG algorithm (𝜃+α)/β during driving segments than those with small speed variability. Several EEG studies have concluded the different roles of the EEG power band in the evaluation of cognitive fatigue (Okogbaa et al., 1994; Lal and Craig, 2002; Zhao et al., 2012). In driving duration, increased alpha activity implies decreased attention, whereas increased theta activity signals the onset of sleep. In contrast, decreased beta activity indicates the decrement of cortical arousal level. The EEG algorithm (𝜃+α)/β is considered as one of the most effective physiological indices to measure the driving fatigue. The smaller EEG algorithm (𝜃+α)/β is, the less fatigue the driver has (Jap et al., 2009). Therefore, the large speed variability group with smaller EEG algorithm (𝜃+α)/β may have less cognitive fatigue. This further proved the suggestion of the dynamic model (Hancock and Warm, 1989): in an underload condition, increasing workload, for example regulating speed variability, can help maintain vigilance and resist cognitive fatigue. Meanwhile, our results also implied that drivers could maintain vigilance through manipulating driving behaviors rather than employing extra cognitive tasks. For example, drivers can spontaneously regulate their vehicle speed variability to increase vigilance. According to Hockey’s (1997) compensatory control model, a driver’s self-regulation of vehicle speed is initiatively based on the task difficulty and the workload. Experienced drivers are able to control the resources of cognition and attention, which help them maintain optimal performance using the speed adjustment strategy. Our study indicated that drivers who used better speed adjustment strategies had lower levels of subjective and physical fatigue.

Consistent with the previous study, our study found that the pupil diameter of participants gradually became smaller, as they became more sleepy and less vigilant during the driving task, indicating that participants’ fatigue states were deepened (Heitmann et al., 2001). However, there were no significant group differences in pupil diameter. The possible reason may be that drivers with larger variability of vehicle speed had more workload of processing visual information, thereby increasing the contractility of pupil diameter that resisted their fatigue.

The present study indicated that among the drivers, some preferred larger variability of vehicle speed, whereas others preferred the smaller one during a prolonged drive. However, drivers with large variability of vehicle speed had less sleepiness, less fatigue, and more vigilance in the driving task. These results were consistent with the dynamic model of stress and sustained attention, indicating that drivers with larger speed fluctuation could increase workload that helps them maintain the optimal arousal level in monotonous highway conditions. Physiological data also proved that drivers with large variability of vehicle speed had a smaller EEG algorithm (𝜃+α)/β. These results proposed that manipulating driving behaviors, like speed variability, could have a positive effect on coping with driving fatigue and maintaining vigilance in the underload condition. Future studies need to combine drivers’ individual differences, road conditions, and the difficulty of driving tasks with the self-regulation strategy to further validate the effect of driving behaviors on cognitive fatigue.

## Ethics Statement

This study was carried out in accordance with the recommendations of the ethical review committee of Liaoning Normal University with written informed consent from all subjects in accordance with the Declaration of Helsinki. The protocol was approved by the ethical review committee of Liaoning Normal University.

## Author Contributions

JM and RC conceived and designed the experiments, and wrote the manuscript. JM and JG were involved in the data collection. JM and HJ were involved in the data analysis. RC and ZY made critical comments and revised the manuscript.

## Conflict of Interest Statement

The authors declare that the research was conducted in the absence of any commercial or financial relationships that could be construed as a potential conflict of interest.
